# Mental health and sleep habits/problems in children aged 34 years: a population study

**DOI:** 10.1186/s13030-021-00213-2

**Published:** 2021-05-20

**Authors:** Fumie Horiuchi, Kentaro Kawabe, Yasunori Oka, Kiwamu Nakachi, Rie Hosokawa, Shu-ichi Ueno

**Affiliations:** 1 Department of Neuropsychiatry, Ehime University Graduate School of Medicine, Shitsukawa, Ehime Toon City, Japan; 2 Center for Child Health, Behavior and Development, Ehime University Hospital, Toon City, Ehime Japan; 3 Center for Sleep Medicine, Ehime University Hospital, Toon City, Ehime Japan

**Keywords:** Mental health, Sleep habits, Circadian rhythm, Sleepiness, Snoring, Preschool children, Child and Adolescent Sleep Checklist, Strengths and Difficulties Questionnaire

## Abstract

**Background:**

Sleep is essential for mental health at all ages, but few studies have investigated the importance of sleep for mental health in early childhood. Therefore, this study examined the association between mental health and sleep habits/problems in children aged 34 years.

**Methods:**

Children aged 3 to 4 years who were living in the community (*n*=415; 211/204 boys/girls) were recruited for this study. Their mental health was assessed using the Strengths and Difficulties Questionnaire (SDQ), and their sleep habits/problems were evaluated using the Child and Adolescent Sleep Checklist.

**Results:**

Based on the total difficulties score of the SDQ, the children were divided into two groups: a poor mental health group (*n*=76) and a control group (*n*=339). In terms of sleep habits, which included total sleep time, bedtime, wake time, and nap conditions, there were no differences between the two groups. Regarding sleep-related problems, however, anxiety before going to sleep (*p*=0.026), circadian rhythm abnormalities (*p*=0.014), and sleepiness during classes outside of naptimes (*p*=0.031) were significantly higher in the poor mental health group than in the control group. Multiple regression analysis showed that poor mental health status was significantly associated with sleepiness and snoring (*p*=0.017 and *p*=0.018, respectively).

**Conclusions:**

The mental health status of 34-year-old children was associated with sleep-related problems, namely sleepiness and snoring. Healthcare providers should pay attention to childrens irregular sleep-wake patterns; moreover, interventions for appropriate sleep hygiene will reduce the psychological burden on both children and their families.

## Background

Sleep is associated with cognitive [1], physical [2], psychomotor [3], and temperament development [4] in children. Healthy sleep is generally defined by adequate sleep duration, appropriate timing, good quality, and the absence of sleep disturbances or disorders [5]. Many studies have confirmed the importance of sleep duration for individual health outcomes. The National Sleep Foundation recommends that newborns (03 months) sleep for 1417h/day, infants (411 months) for 1215h/day, toddlers (12 years) for 1114h/day, and preschoolers (35 years) for 1013h/day [6]. Similarly, the American Academy of Sleep Medicine recommends that infants (411 months) sleep for 1216h/day, children (12 years) for 1114h/day, and children (35 years) for 1013h/day on a regular basis, including naps, to promote optimal health [7].

Sleep duration recommendations can help to determine the normative distribution of sleep duration and are important for surveillance and for informing public policies, interventions, and the general publics healthy sleep behaviors [8, 9]. However, these recommendations do not identify the adequate sleep duration associated with health benefits that is tailored to each child. Moreover, it is important to consider not only sleep duration but also appropriate timing, sleep quality, and the absence of sleep disturbances. Currently, there is awareness of the importance of sleep for the mental health of children and adolescents [10,12].

Mental health problems are strongly related to lower educational achievement, maladaptive personality traits, community disorganization, and a higher number of risky behaviors, such as substance abuse or violence [13]. The worldwide prevalence of mental health problems in children and adolescents ranges from 10 to 20% [14]. Several studies have indicated that the most marked reduction in sleep duration and the highest prevalence of daytime sleepiness occur in adolescents [15, 16]. A cross-sectional study with 11,788 adolescents found that short sleep duration was significantly associated with mental health problems [17]. In terms of preschool children, 2- to 3-year-old children with short sleep duration were found to be more aggressive than their counterparts with long sleep duration, while among 4- to 5-year-olds, those with irregular bedtime showed significantly more aggression and attention problems than those with regular bedtime [18]. A robust long-term association was reported between preschool family irregularity, such as lack of day-to-day family routines, and more sleep problems during childhood as well as shorter sleep duration and later sleep onset measured objectively using actigraphy [19]; moreover, effects were reported of early family irregularity on childrens sleep development across childhood.

Sleep is considered important during early childhood; however, there have been few studies of sleep in children aged 3 to 4 years. Therefore, limited information is available about the association between their mental health and sleep habits/problems. Thus, the aim of this study was to investigate the association between mental health problems and sleep habits/problems in community-dwelling children aged 3 to 4 years.

## Methods

### Participants

The present study was conducted in Toon City, Ehime Prefecture, Japan, which had a population of 34,600 and 234 births in 2016. We included all children who attended 42-month health checkups at a local health center between October 2016 and March 2019.

### Instruments

Participants completed a demographic sheet, the Strengths and Difficulties Questionnaire (SDQ), and the Child and Adolescent Sleep Checklist (CASC). The demographic sheet included questions about basic information, such as the childs age, gender, and whether the child attended nursery school, kindergarten, or stayed at home.

#### Mental health problems

The parents provided information about their childs mental health using the parent version of the SDQ, which describes the childs functioning during the preceding 6 months and is used for the detection of emotional and behavioral problems [20, 21]. It comprises 25 items and is organized into the following five subscales: emotional symptoms, such as having many worries, fears, or headaches; conduct problems, such as having a hot temper, fighting, lying, or cheating; hyperactivity/inattention, such as being overactive, impulsive, and easily distracted; peer relationship problems, such as being solitary, picked on, bullied, or not being liked by other children; and prosocial behavior, such as considering other peoples feelings, sharing with other children, or offering to help others. Each item is rated as follows: 0 = not true, 1 = somewhat true, and 2 = certainly true. The scores of the 20 items of the emotional symptoms, conduct problems, hyperactivity/inattention, and peer relationship problems are summed to generate the total difficulties score, which ranges from 0 to 40 [22].

The cut-off values for the total and subscale scores of the SDQ for age 24 years were established so that approximately 80% of children, whose total difficulties score ranged from 0 to 12, were in a close to average group, 12% of children, whose score ranged from 13 to 15, were in a slightly raised group, 4% of children, whose score ranged from 16 to 18, were in a high group, and 4% of children, whose score ranged from 19 to 40, were in a very high group, as defined by the provisional banding of the SDQ scores for 24-year-old children [23]. In the present study sample (*n*=415), Cronbachs was 0.594.

#### Sleep habits and sleep-related problems

Sleep habits and problems were assessed by asking the parents to complete the CASC [24]. This checklist was designed to identify sleep habits and screen for sleep problems among preschoolers, elementary school children, and high school students. The available language options are English and Japanese. It consists of 12 questions regarding sleep habits and a 24-item checklist that addresses sleep-related problems. Sleep habits, which include total sleep time, bedtime, and wake time on weekdays and weekends; nap frequency; and nap duration over the previous week were obtained from the parent-reported version of the CASC. Regarding the 24 items for sleep-related problems, participants were asked to respond using a 4-point frequency scale in which 0 indicated never/unknown, 1 indicated occasionally (01day per week), 2 indicated sometimes (24 days per week), and 3 indicated always (57 days per week), or they responded I dont know. The CASC scores were subdivided into three categories: pre-sleep domain (6 questions), nighttime domain (12 questions), and daytime domain (6 questions). In the present study sample (*n*=415), Cronbachs was 0.658.

### Procedure

The participants were born between February 2014 and July 2017 and attended health check-ups at age 42 months between October 2016 and March 2019. The consultation rate of health checkups in Toon City is almost 100%. We sent a written explanation of our study and its purpose to the childrens parents along with the non-anonymized questionnaires, namely the demographic sheet, SDQ, and CASC. Those who agreed to participate in our study were asked to bring the completed questi onnaires to the health checkup, and they handed them to the public health nurses, who had received an explanation about the study and understood its purpose.

### Statistical analyses

Continuous variables were expressed as meanstandard deviation (*SD*), and categorical variables were expressed as numbers and percentages. Among the four groups, the very high, high, and slightly raised groups were defined as the poor mental health group. The close-to-average group was defined as the control group. The Mann-Whitney U test was used to compare sleep habits between the poor mental health and control groups. The scores on the three sleep domains (pre-sleep domain, nighttime sleep domain, and daytime domain) were also compared between the two groups using the Mann-Whitney U test. Exact tests were used to compare the percentages of children with each sleep-related problem between the two groups. To elucidate the main factors that affected the childrens mental health, the 24 sleep problems listed in the CASC were entered into a multiple regression analysis, using the backward stepwise method. The close-to-average group was scored 0, the slightly raised group was scored 1, the high group was scored 2, and the very high group was scored 3 as the dependent variable. All tests were two-sided and assumed a 5% significance level. All data were analyzed using SPSS Statistics for Windows, version 22.0 (IBM Corp., Armonk, NY, USA).

### Ethics

This study was approved by the Institutional Review Board of Ehime University Graduate School of Medicine (no.1,607,009). In addition, it was performed with the cooperation of Toon City. Consent to participate was assumed with the submission of the completed questionnaires.

## Results

The participants demographic characteristics and sleep habits are shown in Table1. We sent questionnaires to 633 children aged 34 years who were due to attend health checkups and received questionnaires from 425, but 10 were excluded because of inadequate answers to the SDQ. A total of 415 children (boys: 211, girls: 204) and their parents participated in our study. There were 401 mothers, 13 fathers, and 1 was unknown. The mean age of the children was 42.21.2 months. Of the 415 participants, 144 attended kindergarten, 209 attended nursery school, and 62 stayed at home.

**Table 1:** General and sleep characteristics of 415 children

	MeanSD	Range (minmax )
Age (months)	42.21.2	
Gender (boy/girl)	211/204	
Ways to spend time (n)		
Attending kindergarten	144	
Attending nursery school	209	
Staying at home	62	
Total sleep time (minutes)		
Weekdays	63464	480780
Weekends	63666	420780
Bedtime (hours: minutes)		
Weekdays	21:010:39	19:0023:00
Weekends	21:160:44	19:0024:00
Wake time (hours: minutes)		
Weekdays	6:590:35	3:409:00
Weekends	7:320:46	5:3011:30
Naps		
Frequency (days/week)	4.32.4	07
Duration (minutes)	76.734.4	0180

*SD*standard deviation

### Characteristics of the four groups

Table2 shows the characteristics of the three groups in the poor mental health group and those of the control group. Based on the total difficulties scores of the SDQ, 1.2% of children (*n*=5; 4 boys, 1 girl) were classified as being in the very high group, 3.4% (*n*=14; 9 boys, 5 girls) were in the high group, 13.7% (*n*=57; 34 boys, 23 girls) were in the slightly raised group, and 81.7% (*n*=339; 164 boys, 175 girls) were in the close-to-average group. There were no differences in age, gender, or attending kindergarten or nursery school or staying at home among the four groups (*p*=0.706, 0.167, and 0.889, respectively). After the health check-ups at age 42 months, public health nurses picked up 4 out of 5 children in the very high group, 12 out of 14 in the high group, and 32 out of 57 in the slightly raised group as the subjects of follow-up about developmental disabilities.

**Table 2:** Characteristics of the three groups classified into a poor mental health group and the control group

	Poor mental health group *n*=76			Control group *n*=339	
Total difficulties scores	Very high (1940)	High (1618)	Slightly raised (1315)	Close to average (012)	*p*
Age (months)	42.01.2	42.41.5	42.41.2	42.21.2	0.706^a^
*n* (%)	5 (1.2)	14 (3.4)	57 (13.7)	339 (81.7)	
Gender (boy/girl)	4/1	9/5	34/23	164/175	0.167^b^
Kindergarten, nursery school, home	1, 3, 1	5, 8, 1	23, 25, 9	115, 173, 51	0.889^b^

Meanstandard deviation

^a^Analysis of variance, *p*<0.05, ^b^Exact test, *p*<0.05

### Comparison of sleep habits between the two groups

We divided the four groups into two groupsa poor mental health group and the control groupbased on the cutoff points of the SDQ scores. In terms of sleep habits (Table3), the Mann-Whitney U test indicated that there were no differences in total sleep time, bedtime, or wake time on weekdays and weekends, or in nap frequency or nap duration between the two groups.

**Table 3:** Comparison of sleep habits between the poor mental health and control groups

Sleep habits	Poor mental health group *n*=76	Control group *n*=339	*p*^a^
Total sleep time (minutes)	Mean*SD*	Mean*SD*	
Weekdays	626.985.1	635.757.6	0.801
Weekends	632.287.6	636.560.2	0.629
Bedtime (hours: minutes)			
Weekdays	20:590:41	21:010:38	0.882
Weekends	21:150:46	21:160:43	0.930
Wake time (hours: minutes)			
Weekdays	7:020:35	6:570:35	0.373
Weekends	7:360:47	7:300:46	0.218
Nap			
Frequency (days/week)	4.22.4	4.32.4	0.827
Duration (minutes)	85.634.2	86.934.5	0.595

*SD*, standard deviation

^a^Mann-Whitney U test, *p*<0.05

### Comparison of sleep-related problems between the two groups

Table4 shows the comparison of mental health status, as scored using the total SDQ scores, and three domains of sleep-related problems between the two groups. There was no difference in the pre-sleep domain between the two groups (*p*=0.214). Conversely, the scores on the nighttime and daytime domains of the poor mental health group were significantly higher than those of the control group (*p*=0.008 and *p*<0.001, respectively). Table5 presents the percentages of children with sleep problems as indicated by the CASC and using the exact test. Of the 24 items, three items were significantly different between the two groups: anxiety before going to sleep (I feel anxious or afraid when it is time to go to sleep; 8.5% vs. 2.5%), circadian rhythm abnormalities (The amount of sleep I get varies each night; 18.1% vs. 7.8%), and sleepiness during classes, except for when napping (I get sleepy during class, except for when napping; 9.2% vs. 2.9%). More children in the poor mental health group were anxious, had an abnormality in the circadian rhythm, and were sleepy compared to the control group (*p*=0.026, 0.014, and 0.031, respectively).

**Table 4:** Comparison of sleep-related problems between the poor mental health and control groups

	Poor mental health group	Control group	*p*^a^
	Mean*SD*	Mean*SD*	
Pre-sleep domain	3.832.49	3.302.25	0.214
Nighttime domain	6.523.36	5.223.26	0.008*
Daytime domain	3.692.32	2.571.92	<0.001**

*SD*standard deviation

^a^Mann-Whitney-U test, *p<0.05, **p<0.001

**Table 5:** Frequency of sleep-related problems for factors of the Child and Adolescent Sleep Checklist

		Poor mental health group	Control group	
	Checklist	*n* [57 d/w] / *n* [04 d/w] (%)	*n* [57 d/w] / *n* [04 d/w] (%)	*p*^*d*^
Pre-sleep domain	I drink a caffeinated beverage 3h or less before going to bed.	3/71 (4.1)	18/312 (5.5)	0.778
	I play video games, surf the Internet, or send texts 1h or less before going to bed.	15/58 (20.5)	46/285 (13.9)	0.152
	I avoid going to bed even though it is time to go to sleep.	18/56 (24.3)	53/280 (15.9)	0.092
	I feel anxious or afraid when it is time to go to sleep.	6/65 (8.5)	8/312 (2.5)	0.026*
	I have trouble falling asleep when I am by myself.	47/27 (63.5)	188/130 (59.1)	0.513
	Before I fall asleep, my legs feel uncomfortable, as if I cannot hold them still.	3/63 (4.5)	11/299 (3.5)	0.719
Nighttime domain	I snore.	9/66 (12.0)	27/307 (8.1)	0.266
	My breath sounds as if it is getting caught in my throat.	1/73 (13.5)	6/328 (1.8)	1.000
	I stop breathing while I sleep.	1/67 (1.5)	5/321 (1.5)	1.000
	I toss and turn, or change positions often while I sleep.	44/31 (58.7)	162/168 (49.1)	0.159
	I talk in my sleep.	9/64 (12.3)	37/296 (11.1)	0.838
	I cry out in my sleep and wake up during the night.	2/72 (2.7)	7/326 (2.1)	0.670
	I have scary dreams or cry out during nightmares.	3/70 (4.1)	10/315 (3.1)	0.714
	I sleepwalk.	0/74 (0)	1/332 (0.3)	1.000
	My legs twitch while I sleep.	1/73 (1.4)	4/313 (1.3)	1.000
	I urinate in my sleep.	23/48 (32.4)	73/248 (22.7)	0.095
	I grind my teeth while I sleep.	9/65 (12.2)	23/303 (7.1)	0.155
	I sweat excessively while I sleep.	25/49 (33.8)	74/258 (22.3)	0.051
Daytime domain	The amount of sleep I get varies each night.	13/59 (18.1)	26/306 (7.8)	0.014*
	I feel tired or groggy when I wake up in the morning.	22/53 (29.3)	69/264 (20.7)	0.124
	I skip breakfast.	8/67 (10.7)	20/311 (6.0)	0.203
	I get sleepy during class outside of naptime.	6/59 (9.2)	9/297 (2.9)	0.031*
	I fall asleep during class outside of naptime.	2/65 (3.0)	2/304 (0.7)	0.149
	I fall asleep if I sit still (i.e., watching TV, riding in the car).	13/61 (17.6)	43/291 (12.9)	0.349

d/w days/week

^d^Exact test, *p<0.05, significant results are indicated in bold

### Effects of sleep-related problems on mental health

Multiple regression analysis of mental health status and sleep-related problems showed that mental health status was significantly associated with sleepiness during classes (I get sleepy during class outside of naptime), snoring (I snore), and anxiety before going to sleep (I feel anxious or afraid when it is time to go to sleep; Table6).

**Table 6:** Effect of sleep-related problems on mental health: regression analysis with sleep-related problems as the dependent variable

CASC items	B	*95% CI*		*t*	*p-*value	**
I get sleepy during class outside of naptime.	0.114	0.032	0.195	2.746	0.006	0.160
I snore.	0.072	0.007	0.138	2.167	0.031	0.126
I feel anxious or afraid when it is time to go to sleep.	0.092	0.005	0.178	2.085	0.038	0.122

B=unstandardized regression coefficient; =standardized regression coefficient

Mental health status was transformed into two dummy variables: poor mental health group (total SDQ score>12) vs. control group (total SDQ score<13)

## Discussion

This study focused on the association between mental health problems and sleep habits/problems in children aged 3 to 4 years. Based on our results, the prominent sleep problemsanxiety before going to sleep, abnormality in the circadian rhythm, and sleepiness during classes outside of naptimeswere observed more frequently in those with poor mental health than in control participants. Moreover, their mental health status was significantly associated with sleepiness and snoring.

There were no significant differences in sleep duration, bedtime, wake time, or nap condition between the poor mental health group and the control group. A systematic review of the relationship between sleep duration and health indicators in children and youths aged 517 years showed that shorter sleep duration was associated with adverse physical and mental health, such as excess adiposity, poorer emotional regulation and academic achievement, and a lower quality of life [25]. In terms of the early years (04 years), shorter sleep duration is generally associated with higher adiposity, poorer emotional regulation, impaired growth, more screen time, and a higher risk of injuries, although the evidence is not sufficient and is mixed for cognitive development and physical activity [26]. No difference was found in sleep duration according to mental health status in our study, as the children in both groups slept for more than 10h on average, which is within the range of the National Sleep Foundations recommendation that preschoolers (35 years) sleep for 1013h/day [6, 7].

In our study, there was also no difference in the pre-sleep domain, except for anxiety before going to sleep. Baum et al. reported that adolescents aged 1417 years rated themselves as significantly more anxious, angry, confused, and fatigued, and less vigorous, when they had sleep restriction (6.5h in bed per night for five nights) compared with when they had healthy sleep (10h in bed per night for five nights) [27]. Another randomized trial showed that even modest differences in sleep duration over just a few nights can have significant consequences for daytime functioning, including emotion regulation, short-term memory, working memory, and aspects of attention in children aged 812 years [28]. The relationship between sleep problems and mood has been well established not only in adults but also in younger populations; however, evidence in preschool children is lacking. In addition, anxiety in children is difficult to measure even by their own parents, and fear might be misinterpreted as anxiety. Remarkably little clinical or epidemiologic research has examined the nosology, prevalence, or characteristics of clinically significant anxiety symptoms and disorders in preschool children [29]. However, recent statistics suggest that anxiety and depressive symptoms and disorders can occur earlier in life than previously thought, and the burden is large, with negative impact on childrens social, emotional, and cognitive development [30]. Indeed, anxiety and depression are the second and third leading causes of the total disease burden for 514 years old children, surpassed in burden only by asthma [31]. Depression and anxiety disorders at age 3 each predicted the other at age 6, consistent with findings in school-age children, adolescents, and adults [32, 33]. Anxiety before going to sleep in our study might be one precursor of preschool psychiatric disorders, such as anxiety disorder.

In terms of the daytime domain, circadian rhythm abnormality and sleepiness negatively affected mental health in children aged 3 to 4 years. Sleep/wake patterns are influenced by both environmental and genetic factors that vary across different populations and cultures [34,37]. Adolescent sleep-wake patterns have been associated with several factors, such as pubertal development [38, 39], decreased parental monitoring of bedtime [40], increased demands by the school schedule [37, 41], and changes in the circadian rhythm [42]. Moreover, children in families with lower socioeconomic status were found to exhibit a later rise time, longer time in bed, more nocturnal awakening, and more night-to-night variability in bedtime and time spent asleep [43]. However, in preschool children, there is insufficient data on abnormalities in the circadian rhythm, except regarding neurodevelopmental disorders, such as autism spectrum disorder (ASD) [44,46] and attention-deficit/hyperactivity disorder (ADHD) [47]. Previously, we found that emotional and behavioral problems measured by SDQ were more common in children with ASD compared to typically developing children aged 4 to 16 years [48]. Moreover, toddlers with autistic traits more commonly exhibited bedtime resistance, abnormality in circadian rhythm, and sleepiness outside of naptime than did toddlers without autistic traits [49]. It can be said that behavioral challenges, including disabilities or autistic traits, would be related to sleep problems. In our study, public health nurses picked up 48 out of 76 children with higher total SDQ scores (63.2%) as the subjects of follow-up about developmental disabilities. Children with higher total SDQ scores might be more likely to exhibit autistic traits and sleep problems, including circadian rhythm abnormalities and sleepiness.

The main finding of our study was that sleepiness and snoring were strongly associated with mental health status in children aged 3 to 4 years. Sleepiness is a very common complaint among schoolchildren, and data on its actual prevalence differ from study to study. The varying prevalence of daytime sleepiness depends on the questions used, sample sizes, study area, year the survey was conducted, age, and ethnicity. Moreover, each study can capture only limited aspects of sleepiness, and this must be considered when interpreting the results [50]. Sleepiness is also related to other sleep disturbances, such as narcolepsy [51], sleep-disordered breathing [52], and restless leg syndrome [53]. Moreover, children with ADHD report excessive daytime sleepiness and more sleep problems than do typically developing children [54]. Taurines et al. suggested that psychiatric conditions may be present before the appearance of the first definite ADHD symptoms in a form of pre-comorbidity, characterized by temperament factors, sleep disturbances, symptoms of autism spectrum disorders, and atopic eczema [55]. Meta-analyses by Cortese in 2006 and 2009 demonstrated that children with ADHD exhibit a tendency to be sleepier than controls during the daytime but without reaching pathological levels [54, 56]. Sleepiness in the poor mental health group of the current study might be related to pre-comorbidity of ADHD.

Snoring is the major clinical symptom of obstructive sleep apnea (OSA), which is a condition that is characterized by recurrent episodes of gas exchange abnormalities and repeated arousals that affect between 1 and 3% of 2- to 8-year-old children [57,59]. Children who snore but do not fulfill the criteria for OSA are considered to have primary snoring [60]. Daytime sleepiness, behavioral hyperactivity, learning problems, and restless sleep are all significantly more common in habitual snorers [57, 61, 62]. Poor mental health, daytime sleepiness, and snoring might be related to each other. Sleep disturbances, especially OSA, should be taken into account as a cause of poor mental health in early childhood. In addition, not only OSA but also primary snoring should be considered as a cause of poor mental health in children aged 34 years.

Our findings have several clinical implications. It is difficult for parents, especially first-time parents, to notice abnormalities in their childs circadian rhythm. Parents tend to think that their childrens circadian rhythms are natural and take them for granted; therefore, parents do not consult anyone, and the children and their parents gradually become exhausted. Moreover, an abnormality in the circadian rhythm affects daytime functioning and leads to poor mental health. Once parents can understand that abnormality in circadian rhythm is not because of the way they have raised their children, they will easily accept the existence of sleep disturbances or poor sleep hygiene. Interventions for better sleep hygiene could then be accepted by children and their parents, which would consequently constitute an early intervention for the childrens development. We hope that our findings promote not only good sleep hygiene but also early intervention for mental health and development. However, more research is required to address longitudinal changes in developmental trajectories and sleep habits/problems.

This study had several limitations. First, the CASC was originally developed in Japanese and has served as a valid and effective tool for the assessment of parents perspectives of their children; however, it has not been fully validated. Further research must be conducted to assess the criterion validity of the questionnaire. Second, the cutoff points of the SDQ scores for 24-year-old children were defined in the UK, and Japanese children might have different cutoff points. Indeed, the percentages of the very high and high groups were 1.2 and 3.4%, respectively, which were smaller than 4 and 4% in the UK, respectively [23]. Third, most children attended nursery schools or kindergartens, and the parents did not know how their children spent their day and could not precisely evaluate their childrens activity during the day. Fourth, sleepiness is difficult to assess, especially in children, as they cannot express their subjective sleepiness themselves and tend to be more hyperactive than usual when they are sleepier. Preschool children are notoriously difficult to observe in laboratory settings when using polysomnography for nighttime sleep and the multiple sleep latency test for daytime sleepiness. Fifth, we did not investigate the presence or absence of neurodevelopmental disorders. Therefore, future work might consider using more objective approaches, such as actigraphy or portable sleep study equipment, for sleep evaluation. Moreover, further research must be conducted to assess the criterion validity of the CASC questionnaire. Furthermore, association between sleep-related problems and neurodevelopmental disorders should be addressed.

## Conclusions

This study revealed a strong association between poor mental health and daytime sleepiness and snoring among Japanese children during early childhood. Parents need to pay attention to their childrens sleep-wake patterns, including daytime sleepiness and snoring during sleep. Public health nurses and healthcare providers should intervene to address inadequate sleep hygiene at an earlier stage to promote childrens mental and physical development.

## Data Availability

The datasets used and/or analyzed during the current study are available from the corresponding author on reasonable request.

## Abbreviations

CASC: Child and Adolescent Sleep Checklist
OSA: Obstructive Sleep Apnea
SDQ: Strengths and Difficulties Questionnaire
